# Screening of FDA-Approved Drug Library Identifies Adefovir Dipivoxil as Highly Potent Inhibitor of T Cell Proliferation

**DOI:** 10.3389/fimmu.2020.616570

**Published:** 2021-01-08

**Authors:** Linda Voss, Karina Guttek, Annika Reddig, Annegret Reinhold, Martin Voss, Burkhart Schraven, Dirk Reinhold

**Affiliations:** ^1^ Institute of Molecular and Clinical Immunology, Otto-von-Guericke-University Magdeburg, Magdeburg, Germany; ^2^ Health Campus Immunology, Infection and Inflammation (GC-I3), Otto-von-Guericke-University Magdeburg, Magdeburg, Germany

**Keywords:** apoptosis, DNA damage, inhibitor of T cell activation, adefovir dipivoxil, drug repositioning

## Abstract

Repositioning of approved drugs for identifying new therapeutic purposes is an alternative, time and cost saving strategy to classical drug development. Here, we screened a library of 786 FDA-approved drugs to find compounds, which can potentially be repurposed for treatment of T cell-mediated autoimmune diseases. Investigating the effect of these diverse substances on mitogen-stimulated proliferation of both, freshly stimulated and pre-activated (48 h) peripheral blood mononuclear cells (PBMCs), we discovered Adefovir Dipivoxil (ADV) as very potent compound, which inhibits T cell proliferation in a nanomolar range. We further analyzed the influence of ADV on proliferation, activation, cytokine production, viability and apoptosis of freshly stimulated as well as pre-activated human T cells stimulated with anti-CD3/CD28 antibodies. We observed that ADV was capable of suppressing the proliferation in both T cell stimulation systems in a dose-dependent manner (50% inhibition [IC50]: 63.12 and 364.8 nM for freshly stimulated T cells and pre-activated T cells, respectively). Moreover, the drug impaired T cell activation and inhibited Th1 (IFN-γ), Th2 (IL-5), and Th17 (IL-17) cytokine production dose-dependently. Furthermore, ADV treatment induced DNA double-strand breaks (γH2AX foci expression), which led to an increase of p53-phospho-Ser15 expression. In response to DNA damage p21 and PUMA are transactivated by p53. Subsequently, this caused cell cycle arrest at G_0_/G_1_ phase and activation of the intrinsic apoptosis pathway. Our results indicate that ADV could be a new potential candidate for treatment of T cell-mediated autoimmune diseases. Prospective studies should be performed to verify this possible therapeutic application of ADV for such disorders.

## Introduction

Pharmaceutical drug research and development is a time-consuming and cost-intensive process. It is estimated that development of one single drug costs around US$1.3 billion to US$1.7 billion (1, 2). Usually, it takes 10 to 15 years from compound discovery to approval of a new drug (3). Only 1 out of 1,000 potential compounds passes the pre-clinical phase to human clinical trials. 50% of drugs that reach phase III of clinical testing get no approval (4). Repurposing of established drugs for new indications could be an alternative way to *de novo* drug discovery and development (5). This procedure offers several advantages: development risk and costs are lower due to already available safety and pharmacokinetic profiles (6). Furthermore, the time for drug development can be reduced, because preclinical testing and formulation development has already been done (5). Taken together, the repositioning of known drugs for new applications saves several years development time while lowering risk and costs at the same time (6).

More than 80 different autoimmune diseases are known today (7). Up to 5% of the human population develops autoimmunity which is associated with high healthcare costs, since autoimmune diseases have a high prevalence in the younger population and are often chronic (8, 9). An increasing incidence for autoimmune diseases such as type I diabetes (10), systemic lupus erythematosus (11), rheumatoid arthritis (12) or multiple sclerosis (13) has been observed in the last several years. There is a great variance in the appearance of autoimmune diseases depending on the affected organ and their clinical manifestation (9). However, the critical role of T cells in pathogenesis of autoimmune diseases is well accepted. Central and peripheral immunogenic tolerance prevents T cell reactivity against self-antigens. Though, in autoimmune diseases these control mechanisms are dysregulated (14). Different T cell subsets play a role in disease progression. Usually, regulatory T cells inhibit disease development by tightly controlling autoreactive T cell and B cell responses. Naïve T cells undergo clonal expansion and become activated effector cells after antigen exposure (15). It has been shown, that these activated T cells are of particular importance in chronic autoimmune inflammation (16). However, current treatment strategies often show a number of side effects and limited efficacy (17).

Previously, our group demonstrated that commonly used immunosuppressive drugs, like cyclosporin A and dexamethasone, failed to suppress the proliferation of pre-activated T cells (18). As described above, these activated autoreactive T cells are the key players in the immunopathogenesis and development of various autoimmune diseases. Therefore, it is crucial to find compounds that not only prevent activation of naïve, resting T cells, but are also efficient in suppressing already activated T cells. This dual effect can contribute to improved therapy strategies against autoimmune diseases.

Here, we performed a drug screening of 786 FDA-approved drugs with the aim to find compounds that inhibit proliferation of both, freshly stimulated and pre-activated peripheral blood mononuclear cells (PBMCs). Therefore, we initially developed a screening system. In the first step, we screened for substances, which inhibit proliferation of freshly stimulated PBMCs by stimulating the cells with phytohemagglutinin (PHA) in presence of the compounds. In the next step, we tested the initial hits for their capability to inhibit proliferation of pre-activated PBMCs. For that reason, PBMCs were pre-stimulated with PHA for 48 h. Afterward, the compounds were added to these pre-activated cell cultures. As result of the screening, we revealed Adefovir Dipivoxil (ADV) as a potent drug, which inhibited cell proliferation of both T cell systems in a nanomolar concentration range. ADV is an antiviral drug that belongs to the group of acyclic nucleoside phosphonates (19). It is used for the treatment of chronic hepatitis B, sold under the name Hepsera (20).

## Materials and Methods

### Reagents

Enzo’s SCREEN-WELL® FDA approved drug library V2 was purchased from Enzo Life Sciences, Inc. (Farmingdale, NY, USA) and used for the present in vitro screening study. ADV and Cladribine were purchased from Selleckchem (Houston, USA). Hybridoma supernatants of mouse anti-human CD3ϵ (OKT-3) and CD28 (248.23.2) monoclonal antibodies were produced in our laboratory.

### Cells

Human PBMCs were isolated by Ficoll gradient (Biochrom, Berlin, Germany) centrifugation of heparinized blood collected from healthy volunteers. Human T cells were further purified by non-T cell depletion using the “Pan T cell isolation kit II” (Miltenyi Biotec GmbH, Bergisch Gladbach, Germany). The resulting pan T cell purity was >97%. Cells were washed twice and resuspended in serum-free AIM-V culture medium (Invitrogen, Eggenstein, Germany). The study was approved by the local ethics committee (No. 141/19). All blood donors gave their written informed consent.

### Screening PBMCs Proliferation Assay

Enzo´s SCREEN-WELL® FDA approved drug library V2 (Enzo Life Sciences Inc.) was used to screen for compounds that inhibit proliferation (DNA synthesis) of freshly stimulated PBMCs and pre-activated PBMCs. The library contains 786 compounds at a concentration of 10 mM in DMSO.

First, PBMCs (10^5^ cells/100 µl) were seeded in quadruplicate cultures in 96 well microtiter culture plates (TPP Techno Plastic Products AG, Trasadingen, Switzerland) and incubated with phytohemagglutinin (PHA; 1 µg/ml, Remel Europe Ltd., Dartford, UK) in presence and absence of various concentrations (0.1, 1, or 10 µM) of drug compounds or DMSO as vehicle control. Proliferation of treated cells was determined by a standard [^3^H]-methyl-thymidine ([^3^H]-TdR) incorporation assay. After 72 h, cells were pulsed with [^3^H]-TdR (Perkin Elmer, Boston, MA, USA) at a dose of 0.2 μCi/well for additional 6 h. At the end of the incubation period, cells were harvested, and radioisotope incorporation was measured as an index of lymphocyte proliferation using the betaplate liquid scintillation counter MicroBeta (Wallac, Turku, Finland).

In a second step, the initial hits including ADV were tested for their ability to influence pre-activated PBMCs. Therefore, PBMCs were pre-stimulated with PHA (1 µg/ml). After 48 h, these compounds were added in various concentrations (0.1, 1, or 10 µM). Cell cultures were incubated for additional 24 h at 37°C. After this incubation period proliferation was also assessed by [^3^H]-TdR incorporation.

### T Cell Proliferation Assay

Resting human T cells (10^5^ cells/100 μl) were incubated in quadruplicate cultures in 96-well microtiter culture plates (TPP Techno Plastic Products AG) coated with anti-CD3e (OKT-3) and anti-CD28 (248.23.2) monoclonal antibodies. To investigate the effect on freshly stimulated T cells, increasing concentrations of ADV, Cladribine or DMSO as vehicle control were added in parallel at the beginning of the experiment. In addition, to study the effect on pre-activated T cells, compounds were added 48 h after stimulation. All cell cultures were incubated for 72 h at 37°C. Proliferation was assessed by measuring [^3^H]-TdR incorporation. Therefore, [^3^H]-TdR was added at 0.2 μCi/well for the last 6 h of the incubation. At the end of the incubation period, cells were harvested, and radioisotope incorporation was measured using the betaplate liquid scintillation counter MicroBeta (Wallac). Results were calculated as mean percentage ± SEM of DNA synthesis in relation to control cultures set to 100%.

### CFSE Cell Proliferation Assay

A Cell Trace CFSE (Carboxyfluorescein succinimidyl ester) Cell Proliferation Kit (Invitrogen, Carlsbad, CA, USA) was used to label the resting T cells. Cells were washed with PBS, resuspended in 5 µM CFSE solution in 1 ml phosphate buffered saline (PBS) containing 5% fetal calf serum (FCS) and incubated for 5 min at room temperature in the dark. Then cells were washed twice in 10 ml PBS containing 5% FCS to remove excess CFSE. Stained T cells were resuspended in AIM-V culture medium at a concentration of 1 × 10^6^ cells/ml and seeded in anti-CD3/CD28 antibody coated 24-well plates (Corning Inc., Amsterdam, The Netherlands). To investigate the effect on freshly stimulated T cells, increasing concentrations of ADV, Cladribine or DMSO as vehicle control were added in parallel at the beginning of the experiment. In addition, to study the effect on pre-activated T cells, compounds were added 48 h after stimulation. After an incubation time of 96 h, cells were transferred to polystyrene tubes, washed with FACS staining buffer and incubated with 1 µl of SYTOX AADvanced dead cell stain solution (Thermo Fisher Scientific, Waltham, MA) for 5 min to exclude dead cells. Cells were analyzed by flow cytometry (LSRFortessa, BD Biosciences, Franklin Lakes, NJ, USA).

### Flow Cytometric Analysis of Activation Markers

Activated T cells were treated with various concentrations of ADV or vehicle control for 16 or 48 h. Afterward, the cells were transferred to polystyrene tubes, washed with FACS staining buffer (BioLegend, San Diego, CA), and incubated with APC-labeled anti-CD69 antibodies or PE-labeled anti-CD25 antibodies at 4°C for 30 min. At the end of the incubation period cells were washed with PBS/2 mM ethylenediaminetetraacetic acid (EDTA), resuspended in FACS staining buffer, and analyzed by flow cytometry (LSRFortessa, BD Biosciences, Franklin Lakes, NJ, USA). Data were acquired by FACSDiva 6.0 software (BD Biosciences) and analyzed using the FlowJo software (version 7.6.4, Tree Star Inc., Ashland, OR, USA).

### Cytokine ELISA

For measurement of cytokine secretion, human T cells (10^6^ cells/ml) were seeded in anti CD3/CD28 antibody coated 24-well plates (Corning Inc.) and treated with increasing concentrations of ADV or vehicle control. Supernatants were harvested after 72 h and cytokine concentrations (IFN-γ, IL-5, IL-10, and IL-17) were determined with specific ELISA (Bio-techne Ltd., Minneapolis, MN, USA) according to manufacturer’s instructions. The optical density of each well was determined at 450 and 570 nm (Tecan Safire Microplate Reader, Tecan Group, Männedorf, Switzerland). The concentration of each cytokine was calculated using a standard curve and the measured absorbance.

### Detection of DNA Double-Strand Breaks

For microscopic analysis, immunofluorescence staining of γH2AX foci was performed. Resting T cells were treated with increasing concentrations of ADV, Cladribine or vehicle control for 8 h. Afterward, cells were washed in PBS, pipetted onto silanized glass slides and fixed for 15 min with 2% formaldehyde. After three washing steps in PBS, cells were permeabilized in 0.2% Triton X-100 on ice and blocked with PBS containing 1% bovine serum albumin (BSA). Subsequently, cells were stained with an anti-phosphohistone H2AX mouse monoclonal IgG primary antibody (Millipore, Schwalbach, Germany, clone JBW301) at a dilution of 1:1,000 for 1 h at room temperature. Slides were washed and subsequently incubated for 1 h at room temperature with a 1:2,000 diluted polyclonal goat anti-mouse IgG antibody conjugated to Alexa Fluor 488 (Lifetechnologies, Darmstadt, Germany). After washing, slides were covered with DAPI-containing mounting medium (Medipan, Berlin/Dahlewitz, Germany) and examined using a fully automated γH2AX foci interpretation system (AKLIDES platform; Medipan, Germany). For each sample at least 200 cells were analyzed and the average number of γH2AX foci/cell was determined (21, 22).

For flow cytometry γH2AX measurements, freshly stimulated T cells or pre-activated T cells were treated with increasing concentrations of ADV or vehicle control. After 24 h cells were washed in PBS and fixed for 15 min with 2% PFA. After washing in PBS containing 0.5% BSA an additional fixation followed in 70% ethanol for 20 min. After two washing steps in PBS, cells were permeabilized in 0.2% Triton X-100 on ice and blocked with PBS containing 1% BSA. Subsequently, cells were stained with an anti-γH2AX antibody at a dilution of 1:1000 for 1 h at room temperature. Cells were washed and incubated with a 1:500 diluted polyclonal goat anti-mouse IgG antibody conjugated to Alexa Fluor 488 for 1 h at room temperature. After washing with PBS containing 1% BSA cells were stained for 15 min with 1 μg/ml propidium iodide (PI)/ RNase staining solution (BD Biosciences, Franklin Lakes, NJ, USA). Quantitative analysis of 50,000 cells per sample was performed on a flow cytometer (LSRFortessa BD Biosciences). The data were analyzed using the FlowJo software 7.6.4 (Tree Star Inc., Ashland, OR).

### Apoptosis Measurements

Cell apoptosis was detected using the Annexin V-FITC Apoptosis Detection Kit with PI (BioLegend, San Diego, CA, USA). Briefly, cells were washed with PBS and re-suspended in 1x binding buffer. Then cells (0.5 × 10^6^/100 µl) were incubated with 2.5 μl of Annexin V-FITC and 2.5 μl of PI for 15 min at room temperature in the dark, terminated by addition of 200 μl of 1× binding buffer. Samples were analyzed by flow cytometry using LSRFortessa (BD Biosciences). Early apoptotic cells were defined due to Annexin V positive and PI negative staining. Late apoptotic and non-viable cells were both Annexin V and PI positive. At least 20,000 cells were examined for each sample. The data were analyzed using the FlowJo software (version 7.6.4, Treestar Inc.).

### Flow Cytometric Analysis of Active Caspase-3/7

Caspase-3 and caspase-7 activation was determined using the CellEvent™ Caspase-3/7 Green Flow Cytometry Assay Kit (Thermo Fisher Scientific, Waltham, MA). The membrane-permeable substrate is cleaved by activated caspase-3 or caspase-7 in apoptotic cells enabling the dye to bind to DNA which induces a green fluorescence signal. Cells were incubated with 500 nM CellEvent™ caspase-3/7 green detection reagent in PBS for 30 min at room temperature in the dark. Quantitative analysis of 20,000 cells per sample was performed on a flow cytometer (FACS Calibur, BD Biosciences).

### IncuCyte Caspase-3/7 and Cytotox Assays

IncuCyte S3 Live-Cell Imaging system (Essen Bioscience, Ann Arbor, MI, USA) was used for kinetic monitoring of cytotoxicity and apoptotic activity of ADV in T cells. Therefore, T cells were seeded in 96-well plates (at a density of 3 × 10^4^ cells/well) and ADV or vehicle control were added in various concentrations. For fluorescence-based cell death analysis, IncuCyte™ Caspase-3/7 Green Reagent (Essen Bioscience, 1.6 µM) and IncuCyte™ Cytotox Red Reagent (Essen Bioscience, 250 nM) were added. Thereafter, plates were placed into the IncuCyte S3 Live-Cell Analysis System at 37°C. Every 3 h, images were taken of each well at 10x magnification for 70 h. Images of green and red fluorescence were monitored and automatically quantified by the IncuCyte S3 v2018B software (Essen Bioscience). Images were analyzed for numbers of green objects (caspase 3/7 positive cells) and red objects (Cytotox positive cells) per well.

### Flow Cytometric Caspase-9 Assay

Cells were analyzed using CaspGLOW Fluorescein Active Caspase-9 Staining Kit (Invitrogen, Carlsbad, CA, USA). The caspase-9 inhibitor LEHD-fluoromethylketone conjugated to FITC (FITC-LEHD-FMK) irreversibly binds to active caspase-9 in apoptotic cells. Briefly, T cells in presence of ADV or vehicle control were centrifuged for 5 min at 400*g* and the supernatant was removed by aspiration. The cell pellet was resuspended in 200 μl of PBS, containing FITC-LEHD-FMK at a dilution of 1:500, and the cells were incubated for 30 min at 37°C. Afterward, the cell suspension was centrifuged, the supernatant was removed, and the cells were washed twice in 300 μl of wash buffer. After the final washing step, cells were resuspended in 200 μl of wash buffer, and analyzed using flow cytometry (FACS Calibur, BD Biosciences).

### Western Blot

Cells were lysed using radioimmunoprecipitation assay buffer (RIPA)-based lysis buffer supplemented with Na_3_VO_4_, phenylmethylsulfonylfluorid (PMSF), and complete EDTA-free protease inhibitor mixture (Roche, Basel, Switzerland). Protein concentrations were determined by Pierce BCA Protein Assay Kit (Thermo Fisher Scientific). Equal amounts of protein were separated on sodium dodecyl sulfate (SDS) polyacrylamide gels and transferred to nitrocellulose membrane. Membranes were blocked with 5% non-fat dry milk in tris buffered saline (TBS) containing 0.1% Tween-20 (TBS-T) for 1 h. After blocking, the membrane was incubated with the primary antibody overnight at 4°C. Next, the membrane was washed three times with TBS-T and incubated with the appropriate secondary antibody at a dilution of 1:10,000 for 1 h at room temperature (LI-COR Biosciences, Lincoln, NE, USA). The immunoreactive bands were visualized using an Odyssey infra-red scanner (LI-COR Biosciences). The following primary antibodies were used: mouse anti-β-actin antibody (Sigma-Aldrich, St. Louis, MO, USA), mouse anti-p53 (DO-7) antibody (Thermo Fisher Scientific), rabbit anti-p53 P-Ser15 antibody (Cell Signaling Technology, Danvers, MA, USA), rabbit anti-p21 antibody (Abcam, Cambridge, UK), and rabbit anti-PUMA antibody (Abcam).

### Cell Cycle Analysis

Cells treated with ADV at varying concentrations were centrifuged at 200*g* for 5 min at room temperature. After aspirating the medium, cells were resuspended in cold PBS/1% BSA. The cells were then fixed in 70% ethanol while vortexing and stored at −20°C for 2 h. Before analysis, cells were centrifuged two times at 200*g* for 10 min at room temperature to remove the ethanol. Cells were rinsed twice with PBS/1% BSA and stained for 15 min with 1 μg/ml PI/RNase staining solution (BD Biosciences). The samples were measured by FACSCalibur (BD Biosciences). The fraction of cells at each cell cycle phase was quantified by fitting the experimentally obtained histogram with the Dean-Jett-Fox model in FlowJo software version 7.6.4 software (Treestar Inc.).

### Statistical Analysis

GraphPad Prism software version 7.0 (Graph Pad Software, La Jolla, CA, USA) was used for statistical analysis. Significance levels were calculated by repeated measures of One-Way ANOVA and Dunnett’s Multiple Comparison Analysis Test as post hoc test with 95% confidence interval (α = 0.05). Data were presented as the mean with standard error of the mean (mean ± SEM). Within figures the P values are displayed with asterisks (****P ≤ 0.0001, ***P ≤ 0.001, **P ≤ 0.01, *P ≤ 0.05). The IC_50_ values were calculated with GraphPad Prism software using nonlinear regression (curvefit).

## Results

### Screening of FDA-Approved Compounds Revealed ADV as Promising Candidate, Which Inhibits Proliferation of Freshly Stimulated and Pre-activated PBMCs

We screened a library of 786 FDA-approved drugs to find new therapeutics, which can potentially be repositioned for the treatment of T cell-mediated autoimmune diseases. Our screening strategy aimed at identifying therapeutics capable of inhibiting the proliferation of two cell systems: (i) freshly PHA-stimulated PBMCs, as well as (ii) pre-activated PBMCs.

**Figure 1:**
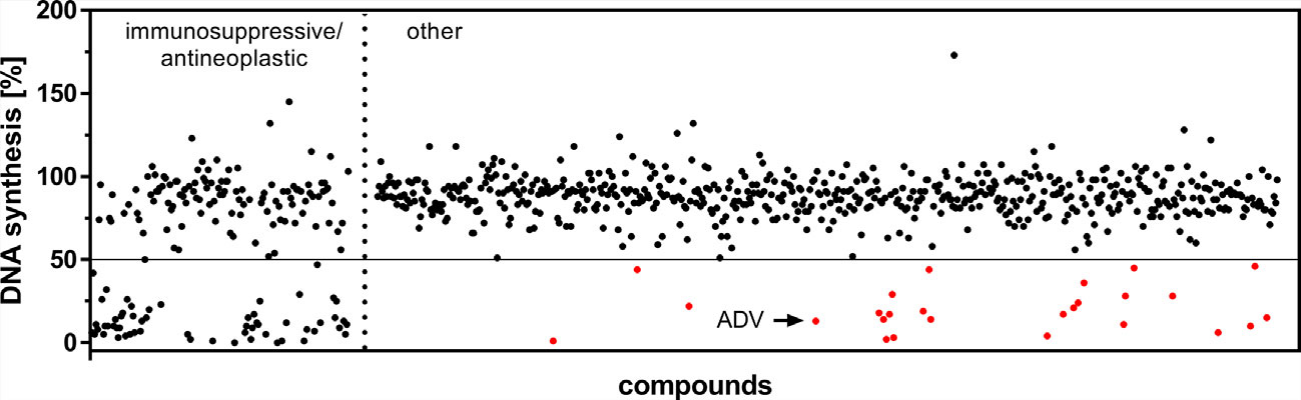
Screening of an FDA-approved drug library. Dot plot of all included 786 compounds screened at a concentration of 1 µM after 72 h to identify substances capable of suppressing DNA synthesis of PHA-stimulated PBMCs. Highlighted are the initial hits (red), comprising compounds which are not FDA-assigned as immunosuppressive or anti-neoplastic drugs (right), but inhibited DNA synthesis >50% (horizontal cut off line).

The first screening step identified 85 compounds (**Figure 1**), which suppressed proliferation of freshly PHA-stimulated PBMCs (>50% inhibition of DNA synthesis determined by standard [^3^H]-thymidine uptake) at a concentration of 1 µM. Out of these 85 hits 59 compounds, already assigned and approved as immunosuppressive or anti-neoplastic drugs, were excluded (**Figure 1**, left).

In a second screening step, the remaining 26 agents were tested for their capability to inhibit proliferation of PBMCs, pre-activated with PHA over a time period of 48 h. Thus, we found 10 compounds, which suppressed the proliferation (>50%) of both freshly stimulated as well as pre-activated PBMCs at a concentration of 1 µM. One of the most potent compounds was ADV, an adenosine monophosphate, which belongs to the class of acyclic nucleoside phosphonates. It was purchased separately to perform additional dose-response studies on isolated human T cells, as described below.

### ADV Inhibited Proliferation and Impaired Activation and Cytokine Production of Freshly Stimulated and Pre-activated T Cells at Low-Nanomolar Concentrations

To confirm the results of the screening obtained from stimulated PBMCs, we next investigated the influence of ADV on proliferation of stimulated human T cells. Cladribine, which is used to treat relapsing-remitting Multiple Sclerosis, was examined in parallel as reference compound. Like ADV, Cladribine belongs to the group of adenosine analogs (23).

Human T cells were stimulated with anti-CD3/CD28 antibodies and cultured with increasing concentrations of ADV or Cladribine for 72 h. For analysis of pre-activated T cells ADV or Cladribine were added to anti-CD3/CD28 stimulated T cells 48 h after stimulation. Proliferation was assessed by measuring [^3^H]-TdR incorporation after 72 h.

**Figure 2:**
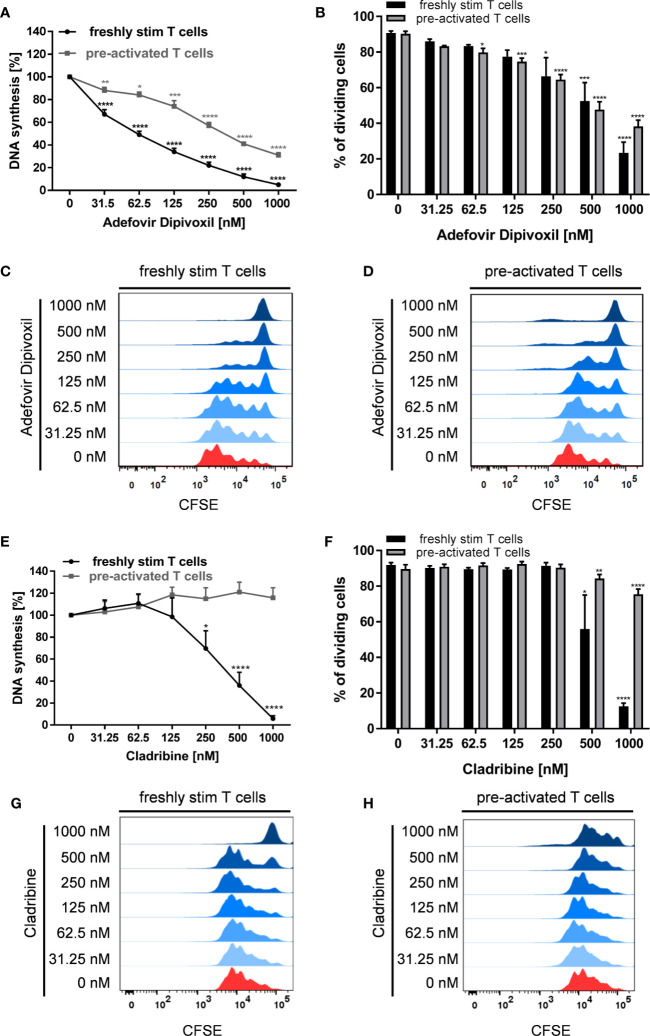
ADV inhibited proliferation of stimulated T cells and pre-activated T cells. **(A–H)** Resting human T cells freshly stimulated (black ) or pre-activated (grey) with anti-CD3/CD28 antibodies were cultured with increasing concentrations of ADV **(A–D)** or Cladribine **(E–H)** for 72 **(A, E)** or 96 h **(B–D, F–H). (A, E)** DNA-synthesis was determined by standard [^3^H]-thymidine uptake. [^3^H]-TdR incorporation is shown as mean percentage ± SEM of DNA synthesis in relation to control cultures (vehicle) set to 100%. **(B, F)** CFSE dilution of stimulated T cells and pre-activated T cells determined as the percentage of dividing cells. Data are presented as the mean ± SEM of n = 3 independent experiments. Histograms of CFSE staining in freshly stimulated T cells **(C, G)** and pre-activated T cells **(D, H)** are shown from a representative experiment.

As expected, ADV treatment strongly suppressed anti-CD3/CD28-mediated proliferation of both, freshly stimulated and pre-activated T cells (**Figure 2A**). Based on [^3^H]-TdR incorporation assay the half maximal inhibitory concentration (IC_50_) values of ADV in freshly stimulated T cells and pre-activated T cells were 63.12 and 364.8 nM, respectively. In contrast, Cladribine significantly suppressed proliferation of freshly stimulated T cells, but it had no effect on pre-activated T cells (**Figures 2E, F**). The IC_50_ value of Cladribine on freshly stimulated T cells was 372.4 nM, while no IC_50_ value was determinable for pre-activated T cells.

In order to analyse how ADV affects T cell division in more detail, we performed CFSE dilution assay (**Figures 2B–D**) to determine the frequency of dividing cells. The percentage of dividing freshly stimulated T cells were strongly reduced from 90.8 ± 1.0% (vehicle control) to 23.4 ± 5.3% in the presence of 1000 nM ADV. Frequency of dividing pre-activated T cells was also significantly lower in samples treated with ADV (from 90.3 ± 1.2% to 38.3 ± 3.0% when treated with 1000 nM ADV). Cladribine reduced the percentage of dividing freshly stimulated T cells only at the two highest concentrations applied (from 91.8 ± 1.1% to 12.6 ± 1.4% when treated with 1000 nM Cladribine). The percentage of dividing pre-activated T cells was slightly reduced in the presence of 1000 nM Cladribine from 89.6 ± 2.0% to 75.5 ± 2.4% (**Figures 2F–H**).

**Figure 3:**
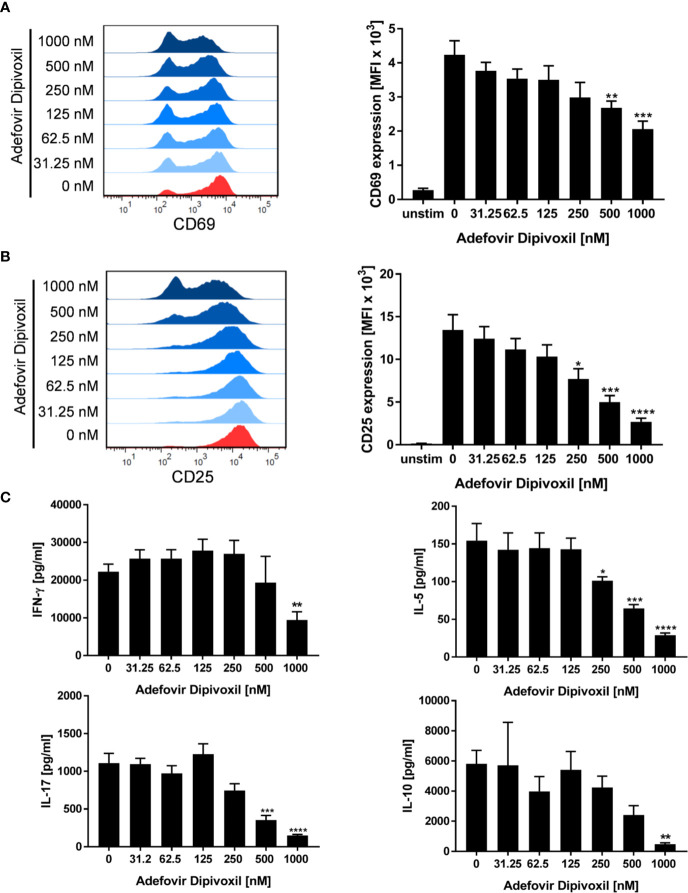
ADV suppresses activation and cytokine production of stimulated T cells. **(A–C)** Resting human T cells were freshly stimulated with anti-CD3/CD28 antibodies and incubated with increasing concentrations of ADV. Expression levels of the T cell activation markers CD69 after 16 h **(A)** and CD25 after 48 h **(B)** were analyzed by flow cytometry. Representative histograms (left) and mean fluorescence intensity (MFI, right) are presented. Cell culture supernatants were harvested 72 h after treatment and concentrations of IFN-γ, IL-5, IL-17, and IL-10 were determined with specific ELISA **(C)**. Data are presented as the mean ± SEM of n = 4 independent experiments. Statistical analysis was performed with One-Way ANOVA and Dunnett’s Multiple Comparison Analysis Test as post hoc test. (****P ≤ 0.0001, ***P ≤ 0.001, **P ≤ 0.01, *P ≤ 0.05).

The ADV-induced inhibition of T cell proliferation was also reflected by a decreased expression of activation markers and a reduced cytokine production. Whereas stimulation of T cells with anti-CD3/CD28 antibodies alone induced the expression of the activation markers CD69 and CD25, treatment with ADV diminished the expression of both activation markers in a concentration-dependent manner (**Figures 3A, B**). Accordingly, the presence of ADV suppressed cytokine secretion of IFN-γ, IL-5, IL-10, and IL-17 (**Figure 3C**) analyzed by ELISA in culture supernatants 72 h after T cell stimulation.

Together, these results clearly show that T cell activation and proliferation can be efficiently suppressed by ADV at low nanomolar concentrations.

### ADV Induced DNA Damage in Resting, Freshly Stimulated, and Pre-ActivatedT Cells

Because ADV is a nucleotide analog that lacks the normal deoxyribose ring, we supposed that ADV is able to induce DNA damage (19). A sensitive biomarker for the detection of DNA double-stranded breaks (DSB) is the phosphorylation of the histone H2AX (γH2AX) that forms nuclear foci at DNA damage sites (24, 25). DSB were quantified by automated microscopic analysis of immunofluorescence-stained γH2AX using the AKLIDES system (21) and by flow cytometry.

**Figure 4:**
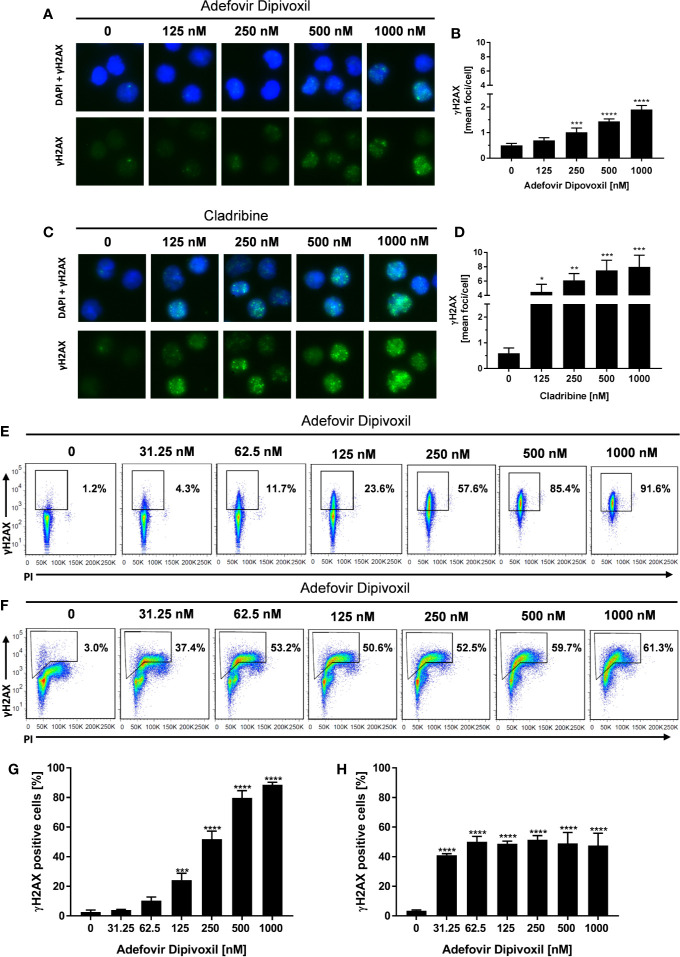
ADV induced DNA double-strand breaks in resting, freshly stimulated, and pre-activated T cells. Resting human T cells were incubated with increasing concentrations of ADV **(A, B)** or Cladribine **(C, D)** for 8 h. The levels of DNA double-strand breaks were assessed by γH2AX immunofluorescence staining and automated foci quantification. Representative microscopic images of γH2AX foci (green), DAPI nuclear staining (blue) **(A, C)** and γH2AX foci quantification **(B, D)** are shown. Each bar represents the mean ± SEM of n = 3 independent experiments. Resting human T cells were freshly stimulated **(E, G)** or pre-activated for 48 h **(F, H)** with anti-CD3/CD28 antibodies and cultured with increasing concentrations of ADV for additional 24 h, respectively. **(E, F)** Representative dot plots of flow cytometric analysis of γ-H2AX and propidium iodide staining of DNA content are shown. **(G, H)** Percentages of γ-H2AX positive cells (mean + SEM) are depicted for 3 independent experiments. Statistical analysis was performed by One-Way ANOVA and Dunnett’s Multiple Comparison Analysis Test as post hoc test. (****P ≤ 0.0001, ***P ≤ 0.001, **P ≤ 0.01, *P ≤ 0.05).

The amount of γH2AX foci was analyzed in unstimulated T cells incubated with ADV or Cladribine as reference compound. Incubation of resting T cells with increasing concentrations of ADV for 8 h caused an increased number of γH2AX foci per cell as shown in **Figure 4A**. ADV led to a significant induction of DNA DSBs, increasing from a mean γH2AX baseline level of 0.50 ± 0.06 foci per cell to 1.90 ± 0.13 foci per cell in presence of 1000 nM of ADV (**Figure 4B**). Treatment of resting T cells with Cladribine for 8 h led to an increase in γH2AX foci formation in a dose-dependent manner, which is illustrated in images taken by the AKLIDES system shown in **Figure 4C**. Focus numbers (**Figure 4D**) were significantly increased, from an average number of 0.60 ± 0.16 foci per cell up to 7.97 ± 1.34 foci per cell when treated with 1000 nM Cladribine.

To study the effect of ADV on DNA damage in more detail, γH2AX expression of activated T cells was analyzed by flow cytometry. Since basal γH2AX level differs according to cell cycle phase, additional assessment of DNA content had to be included. Freshly stimulated T cells treated with 125 nM ADV for 24 h showed a significant increase in γH2AX levels from basal 2.7 ± 1.0% to 24.2 ± 3.8% (**Figures 4E, G**). Expression of γH2AX further increased in a dose-dependent manner reaching 88.6 ± 1.4% after treatment with 1000 nM ADV. As displayed by PI staining, majority of T cells still remained at G_0_/G_1_ cell cycle phase 24 h after activation.

H2AX phosphorylation was also significantly enhanced in pre-activated T cells after exposure to ADV for 24 h. In contrast to freshly stimulated cells the percentage of γH2AX positive cells increased already after treatment with lowest dose of 31.25 nM ADV from 3.5 ± 0.5 to 41.1 ± 0.8%. The histograms revealed an increase in γH2AX primarily in cells with enhanced DNA content (S/G_2_ phase). Due to the heterogeneous cell distribution regarding their cell cycle, the enhanced sensitivity of S/G_2_ phase cells toward ADV-induced DNA damage and an adapted gating strategy, proportions of the population that were γH2AX positive did not change in a concentration-dependent manner and did not exceed 51.4 ± 2.3% in all tested concentrations (**Figures 4F, H**).

Taken together, these results confirm that ADV induced DNA damage in resting T cells, freshly stimulated T cells, and pre-activated T cells.

### ADV-Enhanced Phosphorylation of p53 in Freshly Stimulated and Pre-Activated T Cells

In response to DNA damage, phosphorylation of p53 at Ser15 is one of the key posttranslational modifications of p53. Additionally, its transcriptional activities are significantly induced, which in turn causes cell cycle arrest or apoptosis (26).

**Figure 5:**
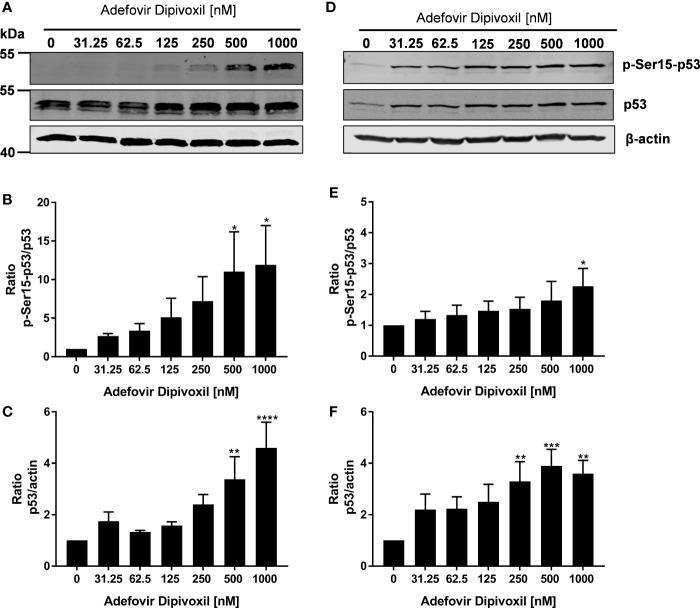
ADV induced phosphorylation of p53 in freshly stimulated and pre-activated T cells. Resting human T cells were freshly stimulated **(A–C)** or pre-activated for 48 h **(D–F)** with anti-CD3/CD28 antibodies and cultured with increasing concentrations of ADV. After additional 24 h incubation, cells were lysed and analyzed by Western blot. **(A, C)** Representative Western blot images of p-Ser15-p53 and total p53, β-actin was used as a loading control. Relative expression of p-Ser15-p53 **(B, E)** and p53 **(C, F)** is shown based on densitometric quantification. Quantitative data are presented as the mean ± SEM from n = 3 independent experiments. Statistical analysis was performed with One-Way ANOVA and Dunnett’s Multiple Comparison Analysis Test as post hoc test. (****P ≤ 0.0001, ***P ≤ 0.001, **P ≤ 0.01, *P ≤ 0.05).

Based on the fact that ADV induced DNA damage, we next analyzed the influence of ADV on p53 phosphorylation by Western blot. After ADV treatment of freshly stimulated T cells for 24 h, a substantial increase in phosphorylation of p53 at Ser15 up to 11.9-fold (for 1000 nM ADV) was observed (**Figures 5A, B**). Interestingly, the expression of total p53 was also elevated up to 4.6-fold in cells treated with 1000 nM ADV compared to untreated controls (**Figure 5C**). In pre-activated T cells only a slight increase of p53 phosphorylation at Ser15 was detectable, which was only significant at the highest ADV concentration applied (2.3-fold at 1000 nM, **Figures 5D, E**). However, ADV again enhanced the levels of total p53 up to 3.6-fold in activated T cells 24 h after incubation with 1000 nM (**Figure 5F**).

### ADV Caused Arrest in G_0_/G_1_ Phase of the Cell Cycle in Freshly Stimulated T Cells

**Figure 6:**
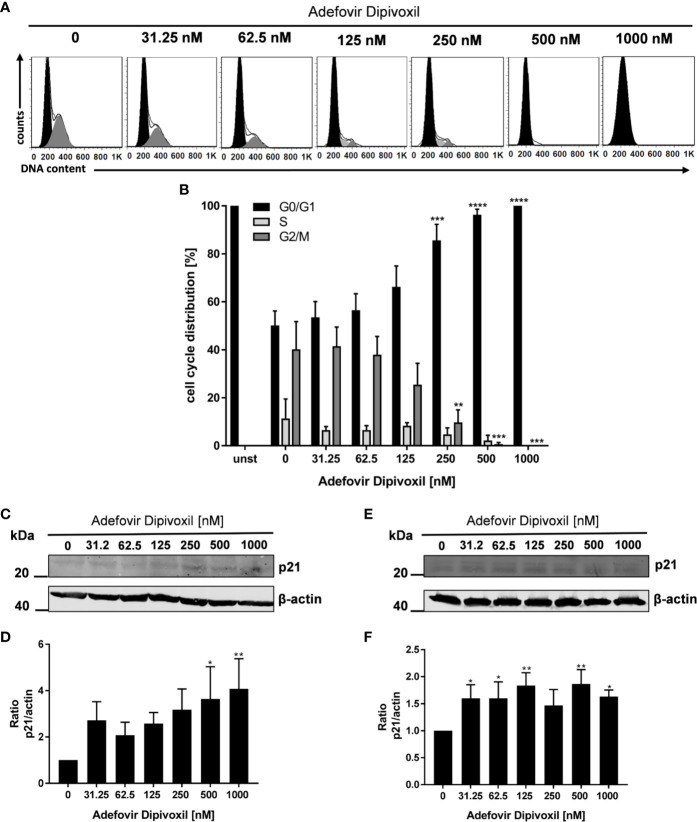
ADV induced cell cycle arrest in G_0_/G_1_ phase by up-regulating p21. Resting human T cells were freshly stimulated with anti-CD3/CD28 antibodies and cultured with increasing concentrations of ADV for 72 h. DNA content and cell cycle phase of cells was determined using propidium iodide staining and flow cytometric analysis. Representative histograms of DNA content are shown in **(A)** (G_0_/G_1_ (black), S (light grey), G_2_/M (dark grey). **(B)** The bars indicate the percentage of cells in G_0_/G_1_ phase, S phase and G_2_/M phase. Data are presented as the mean ± SEM from n = 4 independent experiments. Resting human T cells were freshly stimulated **(C, D)** or pre-activated for 48 h **(E, F)** with anti-CD3/CD28 antibodies and cultured with increasing concentrations of ADV. After additional 24 h cells were lysed and analyzed by Western blot. **(C, E)** Representative Western blot images of p21 expression, β-actin was used as a loading control. **(D, F)** Relative expression of p21 based on densitometric quantification. Quantitative data are presented as the mean ± SEM from n = 5 **(D)**, n = 3 **(F)** independent experiments. Statistical analysis was performed with One-Way ANOVA and Dunnett’s Multiple Comparison Analysis Test as post hoc test. (****P ≤ 0.0001, ***P ≤ 0.001, **P ≤ 0.01, *P ≤ 0.05).

Because it is known that activation of p53 leads to cell cycle arrest or apoptosis, we further investigated possible downstream events (27). Flow cytometry was used to analyse cell cycle distribution following treatment of freshly stimulated T cells with increasing concentrations of ADV for 72 h. As shown in **Figures 6A, B**, we observed a G_0_/G_1_ phase cell cycle arrest in ADV-treated cells. The percentage of T cells in the G_0_/G_1_ phase increased from 50.1 ± 5.3% in control cells to 100% in the presence of 1000 nM ADV, while cell number in G_2_/M decreased from 40 ± 10.0% to 0% under the same conditions, respectively (**Figures 6A, B**). An important downstream target of p53 is p21. This protein inhibits cyclin-dependent kinases, which are required for cell cycle progression (28). Thus, we next analyzed p21 expression by Western blot (**Figure 6C**). ADV treatment of freshly stimulated T cells for 24 h caused a significant increase of p21 expression up to 4-fold (at 1000 nM) (**Figure 6D**). In pre-activated T cells a slight, but significant increase of p21 was observed for almost all concentrations tested (1.6-fold at 1000 nM, **Figures 6E, F**).

These data suggest that ADV induces a cell cycle arrest in G_0_/G_1_ phase in a concentration-dependent manner by up-regulating p21 expression.

### ADV-Induced Apoptosis in Freshly Stimulated and Pre-activated T Cells

**Figure 7:**
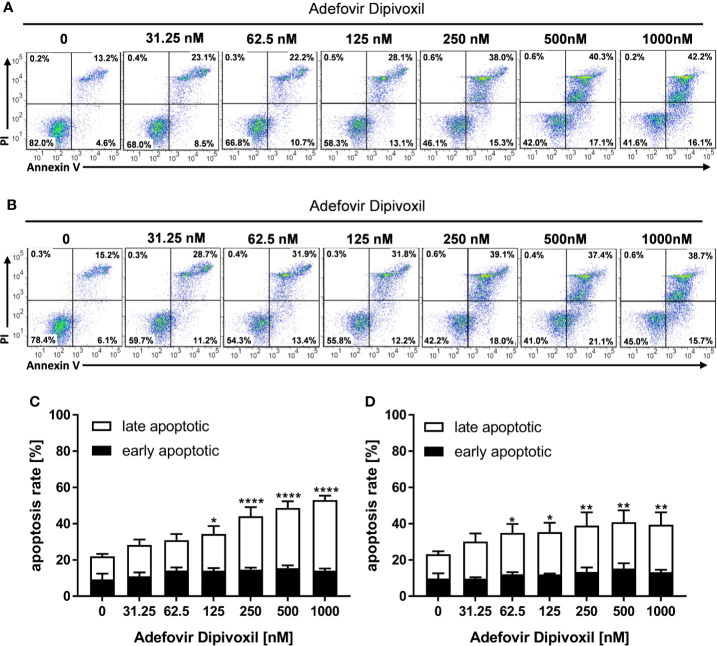
ADV induced cell death in freshly stimulated and pre-activated T cells. Resting human T cells were freshly stimulated **(A, C)** or pre-activated for 48 h **(B, D)** with anti-CD3/CD28 antibodies and cultured with increasing concentrations of ADV. Cells were stained after 72 h with Annexin V-FITC/propidium iodide (PI) for flow cytometric analysis. **(A, B)** Representative flow cytometry dot plots, and **(C, D)** quantification of early and late apoptotic cells of n = 3 independent experiments are shown. Statistical analysis was performed with One-Way ANOVA and Dunnett’s Multiple Comparison Analysis Test as post hoc test. (****P ≤ 0.0001, **P ≤ 0.01, *P ≤ 0.05).

To investigate whether the ADV-mediated activation of p53 leads to cell death, freshly stimulated T cells were incubated with increasing concentrations of ADV for 72 h and subsequently stained with Annexin V-FITC/PI for flow cytometric analysis. In untreated cells, the frequency of apoptotic cells was found to be 22.1 ± 1.0%. In freshly stimulated T cells treated with ADV, the values significantly increased up to 53.0 ± 2.4% apoptotic cells when incubated with 1000 nM ADV (**Figures 7A, C**). In pre-activated T cells treated with 1000 nM of ADV the rate of apoptotic cells significantly increased from 23.2 ± 1.1% up to 39.5 ± 6.7% (**Figures 7B, D**).

**Figure 8:**
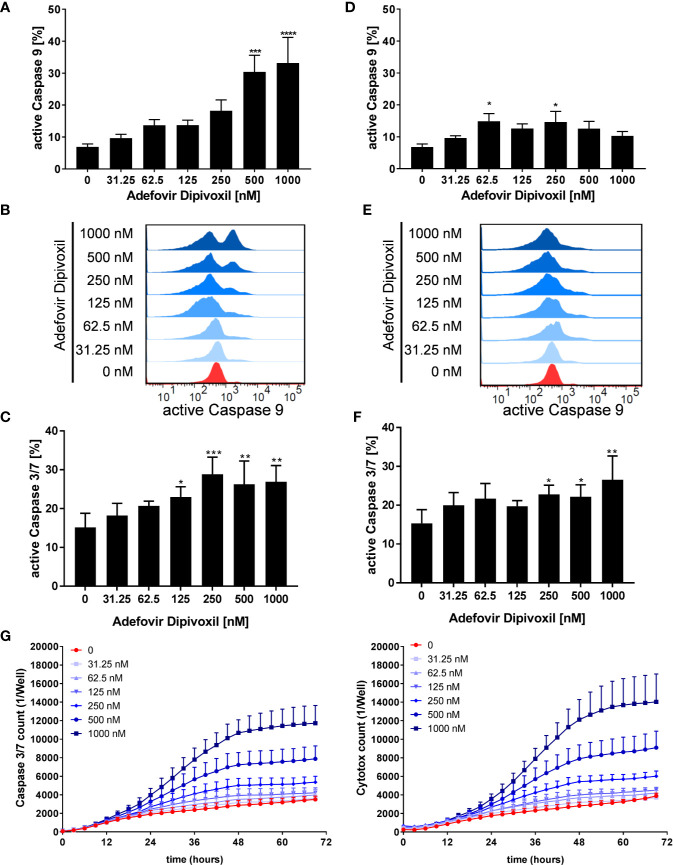
ADV activated the intrinsic apoptosis pathway in freshly stimulated, and pre-activated T cells. Resting human T cells were freshly stimulated **(A–C)** or pre-activated for 48 h **(D–F)** with anti-CD3/CD28 antibodies and cultured with increasing concentrations of ADV. Cells were stained after 72 h with active caspase 9-binding FITC-LEHD-FMK reagent for flow cytometric analysis. Representative histograms **(B, E)** and mean ± SEM percentages of caspase 9 positive cells of n = 4 independent experiments are presented **(A, D)**. Cells were stained after 72 h with CellEvent™ Caspase-3/7 Green detection reagent for flow cytometric analysis. Mean ± SEM percentage of Caspase-3/7–positive cells are shown **(C, F). (G)** For kinetic analyses human T cells were stimulated with anti-CD3/CD28 antibodies and cultured with increasing concentrations of ADV in the presence of Caspase-3/7 reagent and CytoTox Red. Kinetic measures of the number of caspase 3/7 positive cells (left) or CytoTox Red positive cells (right) were recorded by the IncuCyte S3 imaging system at 3 h intervals for 70 h. Data are presented as mean ± SEM from n = 3 independent experiments. Statistical analysis was performed with One-Way ANOVA and Dunnett’s Multiple Comparison Analysis Test as post hoc test. (****P ≤ 0.0001, ***P ≤ 0.001, **P ≤ 0.01, *P ≤ 0.05).

P53 can activate the intrinsic apoptosis pathway (27). Activation of this pathway leads to induction of the initiator caspase 9, which subsequently cleaves downstream caspases, such as caspase 3 or caspase 7 leading to cell death (29). To investigate the activation of initiator caspase 9, freshly stimulated T cells were treated with increasing concentrations of ADV. Flow cytometric analyses showed that ADV treatment enhanced the percentage of caspase 9 positive cells dose-dependently from 6.9 ± 0.8% (untreated) to 33.2 ± 6.9% (1000 nM) after 72 h (**Figures 8A, B**). In pre-activated T cells, incubation with 1000 nM ADV slightly increased the amount of caspase 9 positive cells from 6.8 ± 0.8% in untreated cell cultures up to 14.7 ± 2.9% (**Figures 8D, E**).

Further, the ability of ADV to induce apoptosis was examined flow cytometrically by measuring the activity of caspases 3 and 7 as downstream effectors of the apoptotic signalling cascade. The expression of active caspase 3/7 was significantly increased from 15.1 ± 3.0% (untreated) to 26.9 ± 3.4% (1000 nM) in freshly stimulated T cells after treatment with ADV for 72 h (**Figure 8C**). Moreover, active caspase 3/7 was also elevated in pre-activated T cells after ADV exposure for 24 h from 15.3 ± 2.9% (untreated) to 26.5 ± 5.0% (1000 nM) (**Figure 8F**).

To investigate the kinetics of ADV-induced apoptosis, we next monitored caspase 3/7 activity by IncuCyteS3® live cell imaging, analyzing a specific green fluorescent signal over a period of 70 h (**Figure 8G**, left). ADV induced an increase in caspase 3/7 activity after 24 h, reaching a plateau after 48 h. Additionally, we used Cytotox red staining to determine cell viability over time (**Figure 8G**, right). Adding ADV to freshly stimulated T cells resulted in a dose-dependent decrease of cell viability after 24 h, reaching a maximum after 60 h. Kinetic analyses of caspase 3/7 activity and cell viability confirmed the significant increase in apoptosis determined before by flow cytometric end-point measurements.

**Figure 9:**
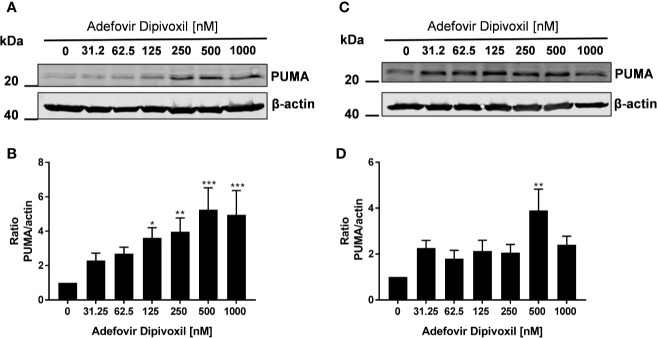
ADV induced up-regulation of PUMA in stimulated T cells. Resting human T cells were freshly stimulated **(A, B)** or pre-activated for 48 h **(C, D)** with anti-CD3/CD28 antibodies and cultured with increasing concentrations of ADV. Cells were lysed after 24 h, respectively, and analyzed by Western blot. **(A, C)** Representative Western blot images of PUMA expression, β-actin was used as a loading control. **(B, D)** Relative expression of PUMA based on densitometric quantification. Quantitative data are presented as the mean ± SEM from n = 6 **(B)**, n = 3 **(D)** independent experiments. Statistical analysis was performed with One-Way ANOVA and Dunnett’s Multiple Comparison Analysis Test as post hoc test. (***P ≤ 0.001, **P ≤ 0.01, *P ≤ 0.05).

Thus, we could show that ADV activates p53 (**Figure 5**) and induced apoptosis in both, freshly stimulated and pre-activated T cells (**Figures 7** and **8**). To gain better insights into the molecular mechanisms involved in ADV-induced apoptosis, we investigate PUMA, which is one of the most important p53 transcriptional targets (27). We treated freshly stimulated T cells with increasing concentrations of ADV and analyzed PUMA expression by Western blot after 24 h. ADV treatment of freshly stimulated T cells caused a significant increase of PUMA expression up to 4.9-fold (at 1000 nM) (**Figures 9A, B**). Results for pre-activated T cells showed a trend for an increased expression of PUMA, which was only significant for cells treated with 500 nM of ADV (3.9-fold, **Figures 9C, D**).

Taken together, these results suggested that ADV induced the intrinsic apoptosis pathway in freshly stimulated T cells as well as in pre-activated T cells by up-regulating PUMA expression.

## Discussion

By screening an FDA-approved drug library, we discovered ADV as a potential drug that could be repositioned for treatment of T cell-mediated autoimmune diseases.

ADV efficiently prevented T cell proliferation in freshly stimulated and pre-activated T cells. Inhibition of proliferation was associated with a decrease in the expression of activation markers CD25 and CD69 and with reduced IFN-γ, IL-5, IL-17, and IL-10 secretion. Importantly, inhibition of cell proliferation by ADV was not only observed in freshly stimulated T cells but also in pre-activated T cells (T cell blasts). This is a clear advantage of ADV, because activated autoreactive T cells are key players in autoimmune diseases (16). We previously showed that not all established immunosuppressive drugs have the capability to suppress proliferation of pre-activated T cells. For example, both, dexamethasone and cyclosporin A failed to inhibit the proliferation of pre-activated T cells (18).

For our study, we chose Cladribine as reference compound. Like ADV also Cladribine is an adenosine analog (30). The drug is widely used as safe and effective treatment for relapsing forms of Multiple Sclerosis (23). However, in our experimental setting Cladribine strongly suppressed proliferation of freshly stimulated T cells, but showed only a weak inhibitory effect on pre-activated T cells (**Figures 2E, F**). Singh et al. reported that Cladribine induced apoptosis in human monocytes, but once differentiated into DC the cells were resistant to Cladribine treatment (31). Our results indicate that ADV might exhibit a higher immunomodulatory potency on T cells compared to Cladribine. Nevertheless, there are some differences in the drug metabolism of both drugs and the effectiveness and resistance of nucleoside analogs is also highly dependent on cell type and cell cycle stage (32, 33).

ADV belongs to the group of acyclic nucleotide phosphonates (34). Previously, it was demonstrated that acyclic nucleoside phosphonates exert immunomodulatory effects. This includes reduced nitric oxid (NO) synthesis in murine macrophages and modulation of cytokine production in macrophages and hepatocytes (35, 36). Furthermore, Zidek et al. (37) showed an anti-proliferative effect of Adefovir in mouse and rat splenocytes. In addition, ADV was shown to be effective against adjuvant-induced arthritis in rats. In this study the authors could demonstrate that ADV exhibits an anti-inflammatory effect not only during the inductive phase of arthritis, but also during the phase of an established disease (38). These observations are in line with our findings, that ADV suppresses both, freshly stimulated T cells as well as pre-activated T cells.

ADV is an ester prodrug, which is converted inside the cell into adefovir, an analog of adenosine monophosphate (39). After cell entry, Adefovir is activated by cellular adenosine monophosphate (AMP) kinases to Adefovir-Diphosphate. This active metabolite has a long intracellular half-life in T cells of approximately 16 h (33). After phosphorylation, Adefovir-Diphosphate acts as an analog of natural deoxynucleoside triphosphates. In this form it serves as substrate and thus as inhibitor of viral polymerases (19). Because Adefovir lacks the normal deoxyribose ring, it acts as chain terminator after incorporation into growing DNA strands. It is known that the active form of Adefovir inhibits not only viral polymerases, but also interacts with human polymerases. Adefovir-diphosphate is also a substrate for DNA polymerases α, β and γ (40). Pisarev et al. could show that 100 µM of active Adefovir is required for complete inhibition of DNA replication (34). This could be one possible mechanism of action, how ADV inhibits the proliferation of T cells.

Chain termination of growing DNA strands could be another consequence of Adefovir incorporation into DNA leading to DNA damage response. Indeed, we found elevated levels of γH2AX in freshly stimulated as well as pre-activated T cells incubated with different concentrations of ADV. In contrast to freshly activated cells, pre-activated cultures contained a heterogeneous T cells population with cells in different cell cycle phases. As also reported elsewhere, cell in S & G_2_ phase already exhibited an elevated background level of γH2AX signals (41). While freshly activated cells, primarily still in G_0_/G_1_ cell cycle phase, only showed enhanced γH2AX signals at higher ADV concentrations, pre-activated T cells, comprising T cells in different cell cycle states, were more sensitive toward ADV, resulting in a significant increase in γH2AX level already at the lowest concentration applied. Consistent with our findings, Khoury et al. demonstrated DNA damage in ADV-treated AML cell lines (42). An elevated γH2AX expression after ADV treatment was also reported in non-small cell lung cancer cell lines (43). Moreover, ADV was found to induce chromosomal aberrations in *in vitro* human peripheral blood lymphocyte assay. However, there was no drug-related increase in tumor incidence in mice and rats (20).

DNA damage response induces a series of events that lead to either cell cycle arrest or in the case of severe damage to apoptosis. P53, a key player in both pathways, is activated by post-translational modifications, like phosphorylation (44). In cell cycle arrest at G_1_ phase, p53 enhances p21 transcription that inhibits cyclin dependent kinase activity (44, 45). We could show that in both ADV-treated freshly stimulated T cells as well as pre-activated T cells phosphorylated and total p53 were upregulated. This indicates an activation of p53 in response to observed DNA damage. Kastan et al. (46) reported an accumulation of p53 protein by agents which cause DNA damage. In agreement with activation of p53, we could observe a cell cycle arrest at G_1_ phase in ADV-treated stimulated T cells.

It is known that p53 directly regulates many genes that induce apoptosis, among them the pro-apoptotic BH3-only members of the BCL-2 protein family, such as PUMA, NOXA, Bad, and BIM (47). These genes enhance the secretion of cytochrome c from mitochondria into the cytoplasm. Cytochrome c activates the adapter protein APAF-1 which is also a p53-regulated gene. This leads to activation of initiator caspase-9 and subsequently to effector caspase-3 and caspase-7 (44). In our study, we could detect enhanced activity of caspase-9 in freshly stimulated T cells treated with ADV. In line with this, ADV induced cell death and activation of caspase-3/7 in freshly activated and pre-activated T cells. These findings indicate that ADV activates the intrinsic apoptosis pathway in freshly stimulated and pre-activated T cells via induction of p53 pathway. Previously, Khoury et al. (42) demonstrated induction of apoptosis including transactivation of pro-apoptotic genes and activation of caspases-9 and -7 in Adefovir-treated AML cell lines.

**Figure 10:**
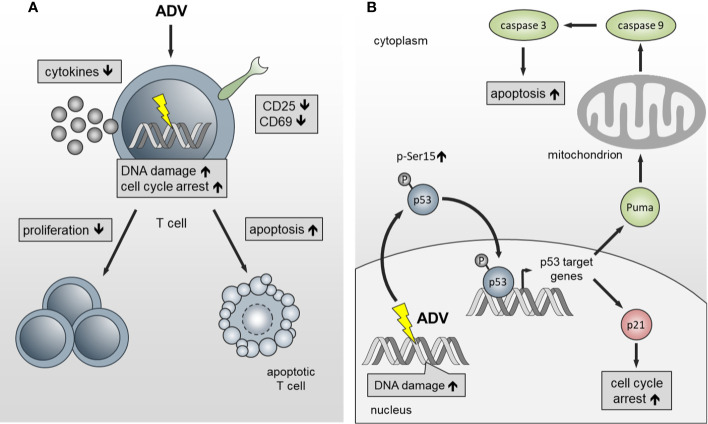
Schematic presentation of the functional effects of ADV on T cells. **(A)** ADV inhibits proliferation in freshly stimulated and pre-activated T cells. Inhibition of proliferation was associated with a decrease in the expression of activation markers and reduced IFN-γ, IL-5, IL-17, and IL-10 secretion. Additionally, DNA damage, cell cycle arrest at G_0_/G_1_ phase and apoptosis was observed. **(B)** ADV treatment induced DNA double-strand breaks (γH2AX foci expression), which led to an increase of p53-phospho-Ser15 expression. In response to DNA damage p21 and PUMA are transactivated by p53. Subsequently, this caused cell cycle arrest at G_0_/G_1_ phase and activation of the intrinsic apoptosis pathway.

In summary, we have identified ADV as potent compound, which inhibits proliferation of both, freshly activated and pre-activated T cells in the nanomolar range. ADV impairs T cell activation and inhibits dose-dependently Th1 (IFN-γ), Th2 (IL-5), and Th17 (IL‑17) cytokine production (**Figure 10A**). In addition, we observed an induction of γH2AX and enhanced phosphorylation of p53. This subsequently led to cell cycle stop at G_0_/G_1_ phase by upregulating p21 and activation of the intrinsic apoptosis pathway in freshly stimulated and pre-activated T cells (**Figure 10B**).

Due to the fact that safety and efficacy of ADV in a daily dosage of 10 mg has been shown in several studies in chronic hepatitis B patients, including patients after kidney transplantation or with renal failure (48), ADV could be a new potential therapeutic candidate for low-dose treatment of T cell-mediated autoimmune diseases. Prospective studies should be performed to verify the possible therapeutic application of ADV for such diseases.

## Data Availability Statement

The raw data supporting the conclusions of this article will be made available by the authors, without undue reservation.

## Ethics Statement

The studies involving human participants were reviewed and approved by Ethics committee of the medical faculty of the Otto-von-Guericke-University Magdeburg. The patients/participants provided their written informed consent to participate in this study.

## Author Contributions

LV designed and performed experiments, analyzed and interpreted the data, and wrote the manuscript. KG and ARed performed experiments and analyzed data. MV analyzed and visualized data. ARei, BS, and DR designed and supervised the research. All authors contributed to the article and approved the submitted version.

## Funding

The project was supported by grants from the European Union Program Regional Development Fund of the Ministry of Economy, Science and Digitalisation in Saxony-Anhalt within the Center of Dynamic Systems and the research alliance “Autonomy in Old Age.”

## Conflict of Interest

The authors declare that the research was conducted in the absence of any commercial or financial relationships that could be construed as a potential conflict of interest.
